# Network pharmacology analysis and cellular experiment validation of puerarin in attenuating TBI through suppression of ferroptosis

**DOI:** 10.1038/s41598-026-53728-5

**Published:** 2026-05-25

**Authors:** Qian Zhang, Guoli Fang, Yafeng Zuo, Qian Niu, Wenlong Zhao, Tingting Zhang, Jincai Li, Zhibing Song

**Affiliations:** 1 School of Traditional Chinese Medicine, Bozhou University, Bozhou, 236800 China; 2 School of Biological and Medical Engineering, Donghua University, Shanghai, 200444 China; 3 School of Chinese Materia Medica, Beijing University of Chinese Medicine, Beijing, 102488 China; 4 Department of Pharmacy, Bozhou Vocational and Technical College, Bozhou, 236800 China

**Keywords:** Puerarin, Ferroptosis, Traumatic brain injury, Network pharmacology, TNF signaling pathway, Computational biology and bioinformatics, Diseases, Drug discovery, Neurology, Neuroscience

## Abstract

**Background: **TBI is a neurological disorder closely associated with ferroptosis. Although puerarin has demonstrated neuroprotective effects, the mechanisms underlying its therapeutic action on TBI via ferroptosis regulation remain unclear. **Objective:** This study aimed to elucidate the potential mechanisms by which puerarin exerts therapeutic effects on TBI, focusing on ferroptosis, through an integrative approach combining network pharmacology, MD simulations, and in vitro experiments. **Methods:** Potential targets of puerarin were predicted using TCMSP, PharmMapper, and SwissTargetPrediction, while ferroptosis- and TBI-related genes were retrieved from FerrDb, OMIM and GeneCards. Differential expression analysis of TBI-related genes was performed using the GSE58483 dataset from the GEO database. GO/KEGG analysis were conducted using the Metascape platform. Molecular docking was carried out with AutoDock Vina and PyMol, followed by MD simulation using Gromacs 2022.6. In vitro experiments were conducted to further validate pathways and targets. **Results:** Key targets, including AKT1, ALB, IL6, and NFKB1, were identified and found to be primarily involved in inflammation-related signaling pathways such as TNF and IL-17. Molecular docking demonstrated stable interactions between puerarin and these targets, which were further supported by MD simulations showing good structural stability and low interaction energies. In HT22 cells subjected to glutamate/erastin-induced ferroptosis, puerarin effectively counteracted ferroptotic cell damage. **Conclusion:** The present investigation reveals that puerarin may inhibit ferroptosis by regulating the TNF signaling pathway, thereby alleviating TBI.

**Supplementary Information:**

The online version contains supplementary material available at 10.1038/s41598-026-53728-5.

## Introduction

Traumatic brain injury (TBI) is a prevalent central nervous system disorder with a rising incidence, particularly in moderate-to-severe cases. It frequently leads to persistent neurological deficits and high disability rates, imposing substantial burdens on healthcare systems and society^1^. Clinically, TBI is characterized by acute cerebral tissue damage followed by secondary neurological impairments^2^. Its pathological progression involves both primary mechanical injury and secondary processes, including oxidative stress, inflammation, mitochondrial dysfunction, and various forms of programmed cell death, all of which exacerbate neuronal damage and impede functional recovery^3,4^.

Ferroptosis, an emerging form of regulated cell death triggered by iron-dependent lipid peroxidation, has garnered increasing attention in TBI research^5^. Accumulation of lipid peroxides and loss of membrane integrity during ferroptosis are considered critical factors contributing to neuronal damage and impaired neurological function^6^. Suppression of ferroptosis has been shown to mitigate secondary brain injury and improve long-term outcomes^7^, highlighting its potential as a therapeutic target in TBI.

Puerarin, a naturally occurring isoflavone derived from *Puerariae Lobatae Radix*, a herb used for medicinal and dietary purposes, exhibits diverse biological activities including anti-inflammatory, antioxidant, and neuroprotective effects, as well as enhancement of cerebral blood flow^8–10^. Evidence suggests that puerarin can attenuate neuronal apoptosis, suppress inflammation, and improve cognitive function in models of ischemic brain injury^11^, Parkinson’s disease^12^, Alzheimer’s disease^13^, and TBI^14^. Additionally, puerarin has demonstrated antioxidant and anti-ferroptotic effects in various disease models, such as osteoarthritis, subarachnoid hemorrhage, and cerebral ischemia-reperfusion injury^15–17^. However, whether puerarin confers neuroprotection in TBI specifically through ferroptosis regulation remains underexplored.

Network pharmacology, integrating bioinformatics and computational simulation methods, elucidates the multi-target mechanisms of traditional Chinese medicine at a systemic level, verifies pharmacodynamic material bases at the molecular level, and interprets the concept of “holistic regulation”^18–20^. In this study, we combined network pharmacology, molecular dynamics (MD) simulations, and in vitro experiments to investigate the interactions between puerarin and ferroptosis, providing novel insights into its neuroprotective mechanisms in TBI. These findings also offer new perspectives for the application of puerarin in central nervous system disorders. The overall research framework is summarized in the Graphical Abstract.

## Materials and methods

### Target identification of puerarin

Using “puerarin” as a keyword, potential targets of puerarin were retrieved from the TCMSP (http://bionet.ncpsb.org/batman-tcm/), SwissTargetPrediction (http://bionet.ncpsb.org/batman-tcm/), and PharmMapper (http://bionet.ncpsb.org/batman-tcm/)^21^. Molecular information of puerarin was obtained from the PubChem database. To further supplement the potential targets, predictive data were collected from SwissTargetPrediction, BATMAN-TCM (http://bionet.ncpsb.org/batman-tcm/), and PharmMapper. The filtering criteria were set as Probability > 0 for SwissTargetPrediction, and Score ≥ 20 with P ≤ 0.05 for BATMAN-TCM. Subsequently, all identified targets were unified using UniProt to ensure consistency and reliability.

### Target identification for ferroptosis and TBI

Genes related to ferroptosis were sourced from FerrDb (https://www.genecards.org/) and GeneCards through keyword-based retrieval using “ferroptosis”^22^. TBI-related genes were searched in GeneCards (https://www.genecards.org/), OMIM (https://www.disgenet.org/), and DisGeNET (https://www.disgenet.org/)^23^ using "traumatic brain injury" as the keyword. A relevance score threshold of ≥15 was applied for GeneCards. Subsequently, deduplication and standardization were performed on all collected gene sets via the UniProt database.

### Construction of the PPI network

Important functional clusters and central genes were detected through the MCODE and cytoNCA plugins, with the top 10 hub genes ranked by network topology chosen as core targets.

Targets intersecting puerarin, ferroptosis, and TBI were extracted using a Venn diagram generated by Venny 2.1. These overlapping targets were analyzed in the STRING database, specifying Homo sapiens and a confidence score cutoff of 0.4 for interactions. Disconnected nodes were removed to construct a high-confidence PPI network. Visualization of the network was performed with Cytoscape 3.10.3 (https://cytoscape.org/)^24^. Important functional clusters and central genes were detected through the MCODE and cytoNCA plugins. Hub genes were identified using the subgraph centrality metric in cytoNCA. All nodes with subgraph centrality > 0 were ranked, and the top 10 genes with the highest subgraph centrality values were selected as core targets for further analysis.

### Validation via gene expression omnibus (GEO) datasets

We retrieved dataset GSE58483 from the GEO database by searching with the keyword “traumatic brain injury” for further analysis. The dataset contains 14 samples (4 healthy controls and 10 TBI samples) and was used as the training set. Data normalization was performed in R, followed by batch effect removal using the “limma” and “pheatmap” packages. Differentially expressed genes (DEGs) were screened with criteria of |log2 fold change| ≥ 2 and adjusted P-value (adj.P.Val) ≤ 0.05. Expression variations were visualized through volcano plots and heatmaps constructed using Bioinformatics.cn platform (https://www.bioinformatics.com.cn/).

### Functional enrichment analysis based on GO and KEGG

Enrichment analysis of core target genes based on GO and KEGG was performed via the Metascape tool (https://metascape.org/)^25^, considering P-values below 0.01 as statistically significant. The top 20 enrichment terms based on −logP values were visualized as bar charts for GO and bubble plots for KEGG using Bioinformatics.cn platform, highlighting major biological functions and pathways.

### Molecular docking

The 2D structure of puerarin was obtained from PubChem and converted into a 3D structure with minimized energy using Chem3D, then saved in mol2 format. Ligand preparation was performed in AutoDock Tools 1.5.7 (https://www.rcsb.org/)^26^ by adding hydrogens and assigning rotatable bonds. The receptor proteins were selected from the top 10 hub targets identified in the PPI network. Their corresponding human protein IDs were retrieved from the UniProt database, and crystal structures were downloaded from the PDB database (https://www.rcsb.org/). Only structures with a resolution lower than 3.0 Å were selected. Protein structures were processed in PyMOL by removing water molecules and native ligands, followed by hydrogen addition and parameterization using AutoDock Tools.

Semi-flexible molecular docking was performed using AutoDock Vina. The docking grid box was centered on the binding pocket defined by the original co-crystallized ligand. The grid center coordinates (X, Y, Z) were set as follows: NFKB1 (8TQD): 5.149, 13.453, −10.752; AKT1 (8R5K): 13.75, −12.916, −19.155; ALB (6YG9): 37.088, −6.587, 17.894; HIF1A (1H2K): 12.1, 13.904, 12.633; IL6 (1ALU): −5.77, −27.919, 10.441; JUN (5FV8): 16.5, 1.241, −17.311; PPARG (1FM9): 12.117, −5.115, 2.691; PTGS2 (5F19): 25.008, 35.663, 41.796; TGFB1 (5VQP): 81.406, 63.067, 10.733; and TNF (2AZ5): −4.865, 85.907, 25.294. Each docking was performed with 50 independent runs to improve sampling accuracy. The conformation with the lowest binding energy was selected as the optimal binding mode. Docking reliability was evaluated by comparing the predicted binding poses with the original co-crystallized ligands. The final docking results were visualized in three dimensions using PyMOL (https://pymol.org/2/) and in two dimensions using LigPlot (https://www.ebi.ac.uk/thornton-srv/software/LigPlus/).

### MD simulation

MD simulations were performed using Gromacs 2020.6 (https://www.gromacs.org/)^27^ for the top four protein–ligand complexes obtained from molecular docking. Protein parameters were assigned using the Amber99SB force field, and ligand topologies were generated with GAFF parameters via Sobtop 1.0 (http://sobereva.com/sobtop/). Each system was solvated in a cubic box filled with SPC water, with a minimum distance of 1.0 nm between the solute and box boundary, and neutralized by adding counterions. Energy minimization was conducted using 10,000 steps of the steepest descent algorithm followed by 10,000 steps of the conjugate gradient method. After sequential equilibration in the NVT and NPT ensembles for 2 ns, a 100 ns production simulation was carried out with a 2 fs integration time step^28^.

The structural stability and conformational dynamics of the protein–ligand complexes were evaluated using Gromacs analysis tools. The analyzed parameters included root mean square deviation (RMSD), root mean square fluctuation (RMSF), radius of gyration (Rg), solvent-accessible surface area (SASA), number of hydrogen bonds, and Gibbs free energy distribution. In addition, binding free energies were calculated using the molecular mechanics/Poisson–Boltzmann surface area (MM/PBSA) method.

### Cell culture

HT22 cells were purchased from Jinyuan Biotechnology (Shanghai, China) and cultured in high-glucose DMEM. The cells were maintained in a humidified incubator at 37°C with 5% CO₂. Although glutamate/erastin-induced HT22 cell injury does not fully replicate the complex pathophysiology of in vivo TBI, this model is widely used to simulate ferroptosis and oxidative stress–mediated neuronal damage relevant to TBI. Therefore, it serves as an appropriate in vitro model for investigating ferroptosis-related neuroprotective effects.

For experimental treatments, cells were first pretreated with the NF-κB inhibitor BAY 11-7082 (5 μM) for 24 h prior to ferroptosis induction. Subsequently, HT22 cells were exposed to glutamate and erastin to induce ferroptosis and were co-incubated with different concentrations of puerarin or ferrostatin-1 (Fer-1, 12.5 μM), a ferroptosis inhibitor. Puerarin, glutamate, erastin, Fer-1, and BAY 11-7082 were purchased from Macklin (Shanghai, China).

### Cell viability assay

Cell viability of HT22 cells was assessed using an MTT kit (Solarbio, Beijing, China). Briefly, 10 μL of MTT solution prepared in DMEM was added to each well of a 96-well plate. After incubation for 4 h at 37°C, the supernatant was carefully removed, and 100 μL of DMSO was added to dissolve the formazan crystals. Absorbance was measured at 450 nm, and cell viability was calculated accordingly.

### Light microscopy and transmission electron microscopy (TEM) observation

HT22 cells were seeded into 6-well plates, and cellular morphology was observed and photographed under a light microscope. For TEM analysis, cells were collected by centrifugation and fixed with 2.5% glutaraldehyde at 4°C for 4 h, followed by 1% osmium tetroxide at 4°C for 2 h. After dehydration and resin infiltration, ultrathin sections (100 nm) were prepared, stained, and examined using a transmission electron microscope.

### Reactive oxygen species (ROS) detection

An ROS detection kit (Solarbio, Beijing, China) was used to measure intracellular ROS levels according to the manufacturer’s instructions. HT22 cells were incubated with serum-free medium containing 10 μL DCFH-DA at 37°C for 20 min in the dark. After washing three times with PBS, the cells were collected, and fluorescence intensity was measured using a fluorescence microplate reader at an excitation wavelength of 504 nm and an emission wavelength of 529 nm.

### Iron content detection

Iron content was measured using a ferrous ion detection kit (Solarbio, Beijing, China) according to the manufacturer’s instructions. Briefly, 5 × 10⁶ cells were homogenized in buffer and incubated with iron assay reagents. Ferrozine chromogenic reagent was then added, and the mixture was incubated at 37°C in the dark for 60 min. Absorbance was measured at 562 nm using a microplate reader, and iron content was calculated accordingly.

### Western blot analysis

HT22 cells were lysed to extract total protein, and the supernatant was collected by centrifugation. Protein concentration was determined using a BCA assay. Equal amounts of protein were separated by SDS-PAGE and subsequently transferred onto PVDF membranes. Based on the molecular weights of the target proteins, the membranes were cut into appropriate sections prior to antibody incubation, and each section was processed independently. Membrane sections were blocked with 5% BSA at room temperature for 90 minutes, followed by overnight incubation at 4 °C with primary antibodies, including anti-GPX4 (1:2000, CST), anti-ACSL4 (1:2000, CST), anti-TNF-α (1:1000, CST), anti-NF-κB (1:1000, CST), anti-IL-6 (1:1000, CST), anti-AKT (1:1000, CST), anti-p-AKT (1:1000, CST), and anti-β-actin (1:3000, CST). After washing with TBST, the membranes were incubated with the secondary antibodies at room temperature for 60 minutes. Protein bands were visualized using an ECL chemiluminescence detection system (Millipore, USA) and imaged with a Chemidoc MP imaging system (Bio-Rad, USA). Band intensities were quantified using ImageJ software.

Due to membrane sectioning prior to antibody incubation, full-length blots were not preserved. All available original, unprocessed images of the membrane sections are provided in the Supplementary Information without selective cropping.

### Statistical analysis

Data are presented as mean ± SD. Statistical analyses were conducted using GraphPad Prism 9.0 software with one-way ANOVA. A P-value less than 0.05 was regarded as statistically significant.

## Results

### Identification of targets associated with puerarin and TBI

**Fig. 1:**
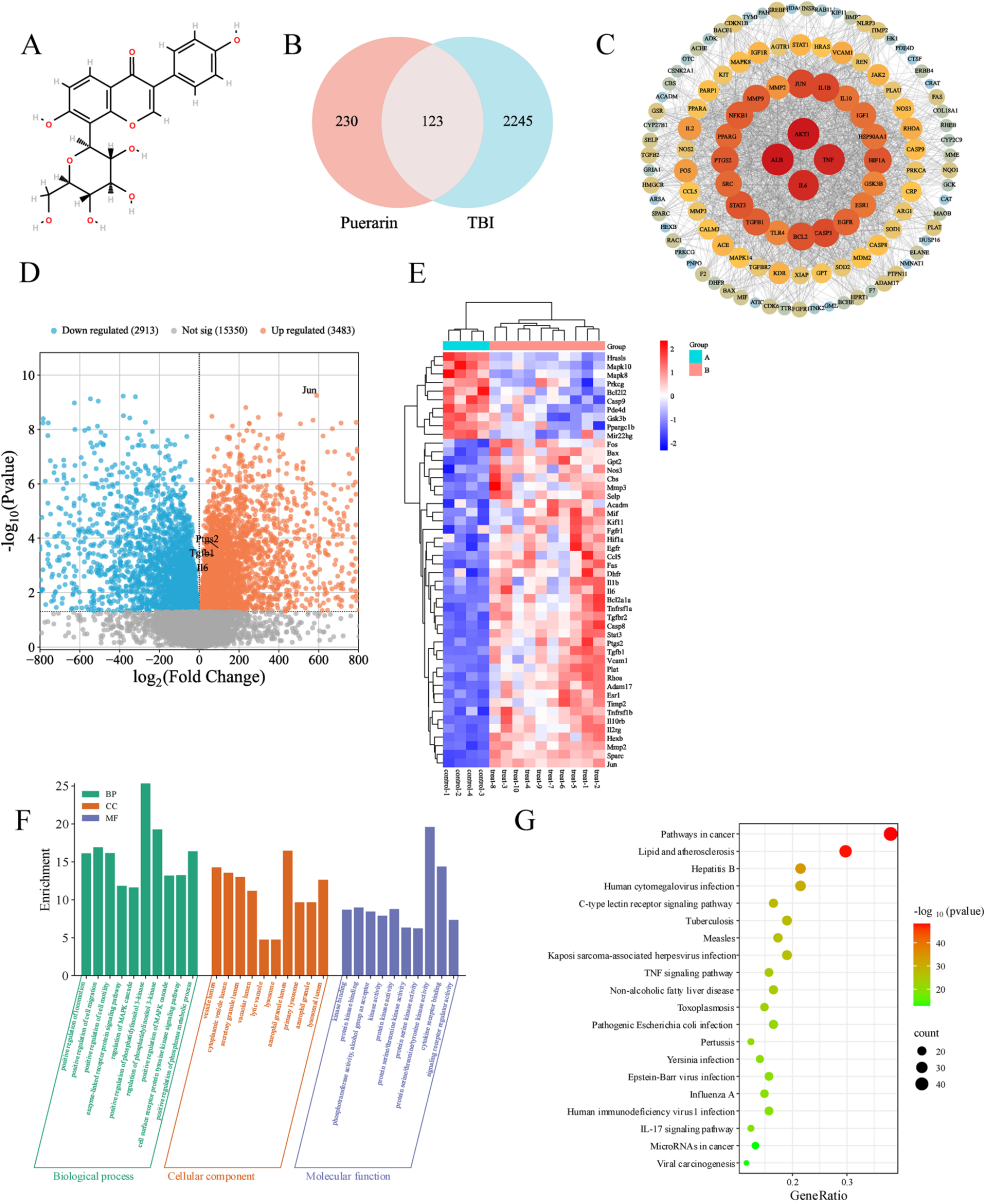
(**A)** Chemical structure of puerarin. (**B)** Venn diagram illustrating the intersection between puerarin-associated and TBI-related targets. (**C)** PPI network of overlapping targets between puerarin and TBI. (**D)** Volcano plot of DEGs from the GSE58483 dataset. (**E)** Heatmap of these DEGs. orange dots denote upregulated genes, blue dots denote downregulated genes, and gray dots denote genes without statistically significant changes. (**F)** Bar chart summarizing the results of GO analysis. (**G)** Bubble plot depicting KEGG analysis outcomes.

The molecular formula of puerarin is C₂₁H₂₀O₉ (Fig. 1A). A total of 466 potential targets were identified from TCMSP, SwissTargetPrediction, and PharmMapper. After removing duplicates and invalid entries, 353 valid targets were retained. TBI-associated targets were collected from the GeneCards and OMIM databases, yielding 2,368 non-redundant disease-associated genes. By intersecting puerarin targets with TBI-related genes using Venny 2.1, a total of 123 common targets were found to overlap between the datasets. Overlapping targets of puerarin and TBI were visually presented through a Venn diagram (Fig. 1B).

### PPI network construction

The PPI network was constructed using 123 overlapping targets and analyzed in STRING with a confidence cutoff of 0.4. The resulting network contained 122 nodes and 856 edges. The interaction data were imported into Cytoscape 3.10.3 for visualization, where node color intensity and proximity to the center reflected the degree of connectivity (Fig. 1C). Nodes with darker colors and more central positions indicate higher importance within the network.

### Validation using GEO dataset

To identify DEGs, gene expression profiles of TBI-injured brain tissues were compared with those of normal brain tissues. Analysis of the GSE58483 dataset identified 6,396 DEGs, including 3,483 upregulated and 2,913 downregulated genes (Fig. 1D). The expression patterns were visualized using a heatmap. Among the 123 intersecting genes between puerarin and TBI, 10 were significantly downregulated and 38 were upregulated. Notably, JUN, IL6, PTGS2, and TGFB1 exhibited marked differential expression (Fig. 1E).

### GO/KEGG analyses

GO enrichment analysis of the 123 putative targets was performed using the Metascape platform. A total of 6,285 significantly enriched GO terms were identified, including 5,094 Biological Process (BP), 411 Cellular Component (CC), and 780 Molecular Function (MF) terms. For clarity, Fig. 1F shows the top 10 enriched terms in each category selected from the top 20 ranked by −logP. The results suggest that puerarin may regulate biological processes such as cell migration, promotion of cell motility, and modulation of the MAPK cascade. At the subcellular level, target proteins were mainly localized to the cytoplasmic vesicle lumen, secretory granule lumen, and lysosomal lumen. In terms of molecular function, targets were primarily enriched in kinase binding, phosphotransferase activity, and protein kinase activity.

KEGG enrichment analysis identified 275 significantly enriched pathways, involving multiple disease- and inflammation-related signaling cascades, including pathways in cancer, the TNF signaling pathway, and lipid metabolism and atherosclerosis (Fig. 1G). These findings indicate that the therapeutic effects of puerarin may be mediated through regulation of these critical pathways.

### Identification of ferroptosis-related genes and integrated analysis with puerarin and TBI targets

#### Overlapping targets and PPI network construction

**Fig. 2:**
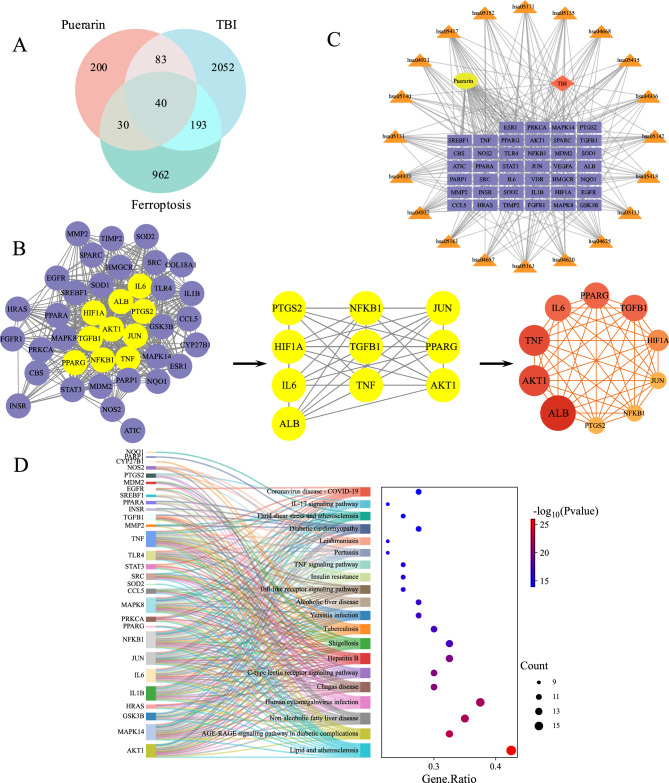
**(A)** Venn diagram illustrating the intersection among puerarin targets, TBI-related genes, and ferroptosis-associated genes. (**B)** PPI network of the 40 overlapping targets. (**C)** Puerarin–Target–Signaling Pathway–TBI interaction network. (**D)** Top 20 KEGG pathways enriched in the overlapping targets involved in puerarin-mediated ferroptosis regulation for TBI treatment.

Using “ferroptosis” as the keyword, ferroptosis-associated genes were retrieved from GeneCards and FerrDb databases. After merging and removing duplicate entries, 1,225 ferroptosis-related genes were obtained. These genes were intersected with the overlapping targets of puerarin and TBI using the Venny 2.1.0 online tool, resulting in 40 common targets (Fig. 2A). These targets were considered potential therapeutic targets through which puerarin may regulate ferroptosis in the treatment of TBI.

The 40 shared targets were uploaded to STRING to construct a PPI network. The resulting network consisted of 40 nodes and 212 edges, with an average node degree of 24.4. In this network, node size corresponds to centrality and potential importance as a key target, with higher degree values indicating greater centrality.

For visualization and topological analysis, the interaction data were imported into Cytoscape (v3.10.3). Network visualization and topological analysis were performed using the cytoNCA plugin. Based on centrality parameters, the top 10 hub genes were identified as ALB, TNF, AKT1, IL6, PPARG, TGFB1, HIF1A, JUN, PTGS2, and NFKB1 (Supplementary Table S1 and Fig. 2B). These genes may play critical roles in the mechanism by which puerarin regulates ferroptosis to alleviate TBI.

Furthermore, an integrated interaction network comprising puerarin, 40 common targets, and 20 KEGG pathways associated with TBI was constructed using Cytoscape (Fig. 2C). This network illustrates the potential mechanisms by which puerarin regulates ferroptosis and modulates TBI-related biological processes. In the network, purple squares represent target proteins, orange triangles denote signaling pathways, yellow ellipses represent puerarin, and reddish-orange diamonds indicate TBI.

#### GO/KEGG analysis

GO enrichment analysis of the 40 potential targets was performed using the Metascape platform, identifying a total of 4,212 enriched terms, including 3,546 BP, 233 CC, and 433 MF terms. Supplementary Figure S1 presents the top 20 significantly enriched terms in each category. The results suggest that puerarin may participate in biological processes such as cell migration and promotion of cell motility. The enriched cellular components mainly include transcription regulator complexes and euchromatin regions, while the molecular functions are primarily associated with transcription factor binding, kinase binding, and nuclear receptor activity.

KEGG enrichment analysis identified 218 significantly enriched signaling pathways. Based on the number of enriched genes (≥34) and ranked by P value, a bubble plot was generated to visualize the top pathways (Fig. 2D). In the plot, larger and redder bubbles indicate higher statistical significance. Notable enrichment was observed in pathways related to lipid and atherosclerosis, as well as TNF and IL-17 signaling, suggesting that puerarin may exert therapeutic effects by modulating inflammation- and metabolism-related pathways. Although the top 20 pathways did not include a canonical ferroptosis pathway, several pathways—such as lipid metabolism, TNF signaling, and oxidative stress–related pathways—are closely associated with ferroptosis regulation. These findings suggest that puerarin may influence ferroptosis indirectly through these mechanisms (Table 1). KEGG pathway enrichment analysis was conducted using the KEGG database (https://www.kegg.jp/)^29^.

**Table 1:** Basic information of the top enriched KEGG pathways.

ID	Term	Enrichment	Pvalue	Count
hsa05417	Lipid and atherosclerosis	59.59	1.29482E-26	17
hsa04933	AGE-RAGE signaling pathway in diabetic complications	97.45	3.16262E-23	13
hsa04932	Non-alcoholic fatty liver disease	67.51	1.15708E-22	14
hsa05163	Human cytomegalovirus infection	50.25	2.64246E-22	15
hsa05142	Chagas disease	88.21	6.37594E-21	12
hsa04625	C-type lectin receptor signaling pathway	86.53	8.12367E-21	12
hsa05161	Hepatitis B	60.38	2.07365E-20	13
hsa05131	Shigellosis	39.37	5.96459E-18	13
hsa05152	Tuberculosis	49.92	7.41814E-18	12
hsa05135	Yersinia infection	60.35	2.42037E-17	11
hsa04936	Alcoholic liver disease	57.83	3.91286E-17	11
hsa04620	Toll-like receptor signaling pathway	69.46	1.85124E-16	10
hsa04931	Insulin resistance	69.46	1.85124E-16	10
hsa04668	TNF signaling pathway	63.62	4.57832E-16	10
hsa05133	Pertussis	87.36	7.93439E-16	9
hsa05140	Leishmaniasis	86.25	8.94626E-16	9
hsa05415	Diabetic cardiomyopathy	40.62	2.03206E-15	11
hsa05418	Fluid shear stress and atherosclerosis	53.70	2.60464E-15	10
hsa04657	IL-17 signaling pathway	71.73	5.03257E-15	9
hsa05171	Coronavirus disease - COVID-19	34.99	1.29482E-26	17

Among the enriched KEGG pathways, “Lipid and Atherosclerosis” and “TNF signaling pathway” were selected for visualization because of their key roles in ferroptosis and TBI pathophysiology, where lipid dysregulation and TNF-driven inflammation promote ROS accumulation and neuronal ferroptosis. Supplementary Figure S2 illustrates the potential puerarin-targeted genes within these pathways, with red-labeled nodes representing predicted targets of puerarin.

### Molecular docking

To further validate the regulatory effect of puerarin on ferroptosis in the treatment of TBI, molecular docking was performed between puerarin and the 10 hub targets: AKT1 (PDB ID: 8R5K), ALB (PDB ID: 6YG9), HIF1A (PDB ID: 1H2K), IL6 (PDB ID: 1ALU), JUN (PDB ID: 5FV8), NFKB1 (PDB ID: 8TQD), PPARG (PDB ID: 1FM9), PTGS2 (PDB ID: 5F19), TGFB1 (PDB ID: 5VQP), and TNF (PDB ID: 2AZ5).

**Fig. 3:**
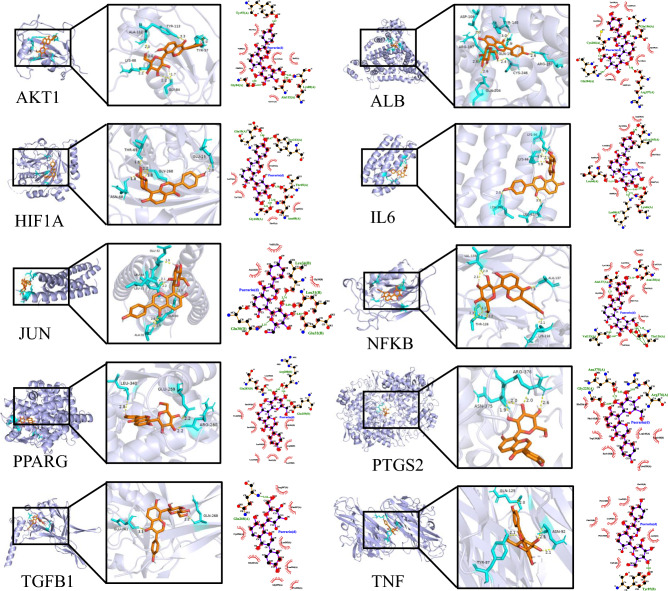
Schematic representations of protein–ligand interactions between puerarin and key targets (AKT1, ALB, HIF1A, IL6, JUN, NFKB1, PPARG, PTGS2, TGFB1, and TNF). For each complex, three visualizations are presented from left to right: overall structure of the complex; three-dimensional receptor–ligand interaction model; two-dimensional receptor–ligand interaction map.

As summarized in Table 2, puerarin showed favorable binding affinities with all selected targets. Notably, the binding energies of puerarin with AKT1, ALB, IL6, NFKB1, and TNF were all below −6.0 kcal/mol, indicating strong binding interactions and stable complex formation (lower binding energy reflects higher affinity and greater complex stability)^30^. Specifically, puerarin–AKT1 formed five hydrogen bonds involving Tyr57, Lys88, Ala112, Gly84, and Tyr113. The puerarin–ALB complex formed seven hydrogen bonds with Cys246, Gln196, Gln204, Arg197, Arg257, Asp108, and Tyr148. Puerarin–IL6 formed four hydrogen bonds with Lys66, Lys86, Leu62, and Leu165. Puerarin–NFKB1 formed four hydrogen bonds with Val133, Ala137, Lys116, and Thr126. Puerarin–TNF formed three hydrogen bonds with Tyr87, Gln125, and Asn92. The interaction models are shown in Fig. 3. These results suggest that the AKT1–puerarin, ALB–puerarin, IL6–puerarin, and NFKB1–puerarin complexes may represent key molecular interactions underlying the ferroptosis-regulating and neuroprotective effects of puerarin in TBI.

**Table 2:** Molecular docking analysis between puerarin and key protein targets.

Proteins	PDB	Binding energy(kcal/mol)	Amino acid residue
AKT1	8R5K	−7.73	ALA-112; TYR-113; LYS-88; GLY-84; TYR-57
ALB	6YG9	−6.53	ARG-257; CYS-246; GLN-204; GLN-196; ARG-197; ASP-108; TYR-148
HIF1A	1H2K	−5.14	THR-65; GLY-268; GLU-19; ASN-68
IL6	1ALU	−6.55	LYS-86; LYS-66; LEU-165; LEU-62
JUN	5FV8	−4.60	GLU-32; LEU-34; LEU-31; ALA-32; GLN-30
NFKB1	8TQD	−7.35	VAL-133; ALA-137; LYS-116; THR-126
PPARG	1FM9	−5.63	LEU-340; GLU-259; ARG-280
PTGS2	5F19	−4.94	ARG-376; ASN-375
TGFB1	5VQP	−3.70	GLU-261; GLN-268
TNF	2AZ5	−6.48	GLN-125; ASN-92; TYR-87

### MD simulation

**Fig. 4:**
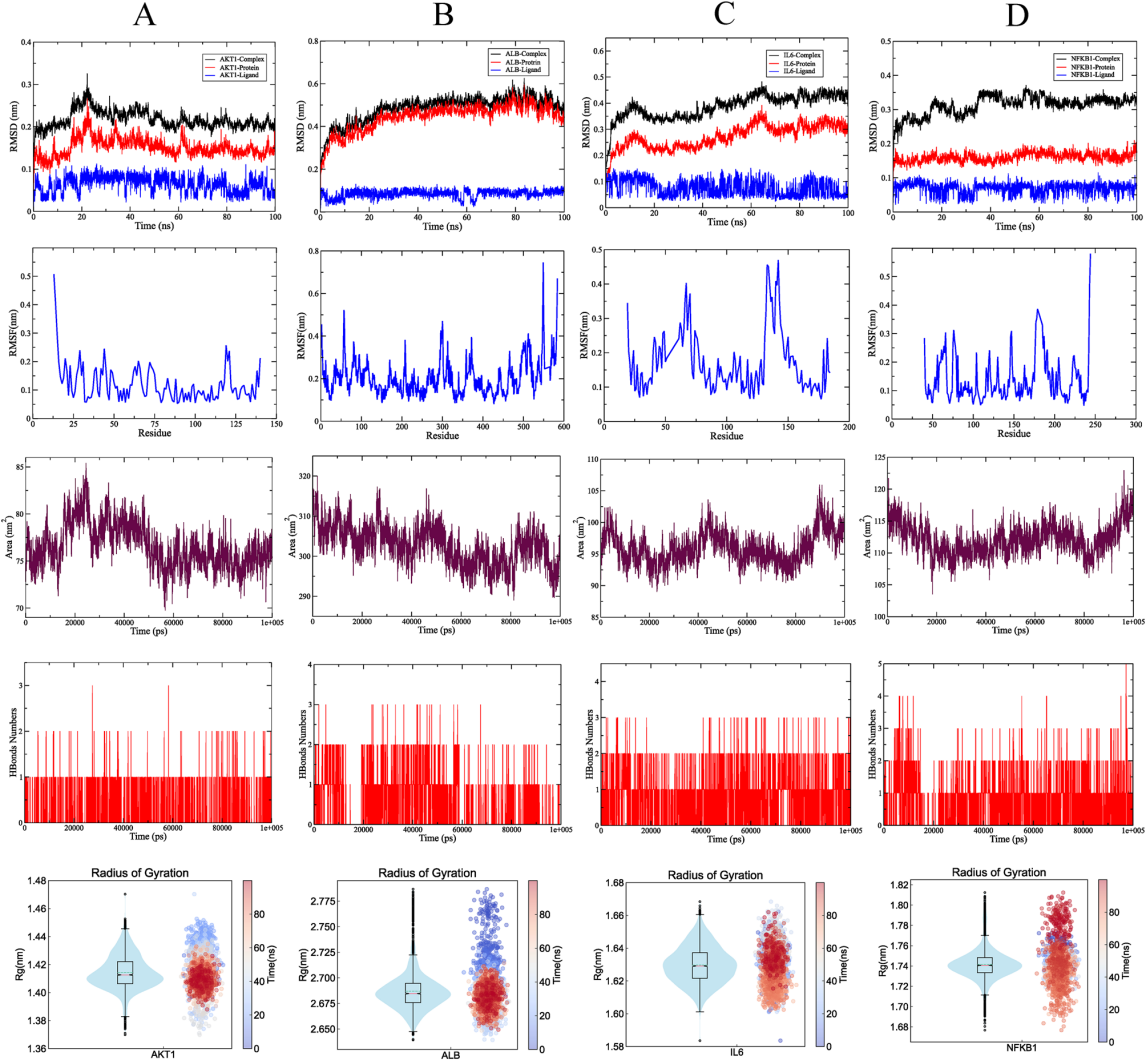
Results of molecular dynamics simulations. (**A)** AKT1–puerarin complex. (**B)** ALB–puerarin complex. (**C)** IL6–puerarin complex. (**D)** NFKB1–puerarin complex.

To evaluate the structural stability of the protein–ligand complexes, the four top-scoring complexes based on binding affinity (AKT1–puerarin, ALB–puerarin, IL6–puerarin, and NFKB1–puerarin) were subjected to 100 ns MD simulations. Key structural parameters and binding stability were subsequently analyzed (Table 3 and Fig. 4A–D).

**Table 3:** Average values and ranges of RMSD, RMSF, Rg, and SASA for each protein–puerarin complex during the 100 ns molecular dynamics simulation.

S. nos	Phytochemicals	Average RMSD (nm)	Average RMSF (nm)	Average Rg (nm)	Average SASA (nm^2^)
1	Puerarin-AKT1 complex	0.21±0.02	0.12±0.06	1.41±0.01	75.72±2.14
2	Puerarin-ALB complex	0.48±0.06	0.20±0.08	2.68±0.02	297.99±6.50
3	Puerarin-IL6 complex	0.38±0.04	0.17±0.09	1.63±0.01	96.01±2.57
4	Puerarin-NFKB1 complex	0.31±0.03	0.13±0.08	1.74±0.02	113.78±2.74

RMSD was used to assess overall conformational fluctuations during the simulations. The RMSD values for AKT1–puerarin, ALB–puerarin, IL6–puerarin, and NFKB1–puerarin were 0.21 ± 0.02 nm, 0.48 ± 0.06 nm, 0.38 ± 0.04 nm, and 0.31 ± 0.03 nm, respectively, indicating that all complexes maintained stable conformations throughout the simulation period^31^.

RMSF analysis revealed average residue fluctuations of 0.12 ± 0.06 nm (AKT1–puerarin), 0.20 ± 0.08 nm (ALB–puerarin), 0.17 ± 0.09 nm (IL6–puerarin), and 0.13 ± 0.08 nm (NFKB1–puerarin). These relatively low fluctuation values suggest stable backbone conformations across the entire protein chains^32^.

Rg, which reflects protein compactness^33^, was calculated as 1.41 ± 0.01 nm for AKT1–puerarin, 2.68 ± 0.02 nm for ALB–puerarin, 1.63 ± 0.01 nm for IL6–puerarin, and 1.74 ± 0.02 nm for NFKB1–puerarin. All complexes exhibited stable Rg trends during the simulation, with no evidence of structural expansion or unfolding.

SASA analysis yielded average values of 75.72 ± 2.14 nm^2^ (AKT1), 297.99 ± 6.50 nm^2^ (ALB), 96.01 ± 2.57 nm^2^ (IL6), and 113.78 ± 2.74 nm^2^ (NFKB1). The relatively narrow fluctuation ranges indicate stable interactions between the protein–ligand interface and the solvent environment^34^, further supporting reliable binding.

Hydrogen bond analysis was performed to evaluate the stability of ligand–protein interactions^35^. Among the four complexes, puerarin formed the highest average number of hydrogen bonds with IL6 (0.79 ± 0.73), followed by NFKB1 (0.72 ± 0.81), ALB (0.51 ± 0.68), and AKT1 (0.23 ± 0.46), indicating sustained intermolecular interactions throughout the simulation.

**Fig. 5:**
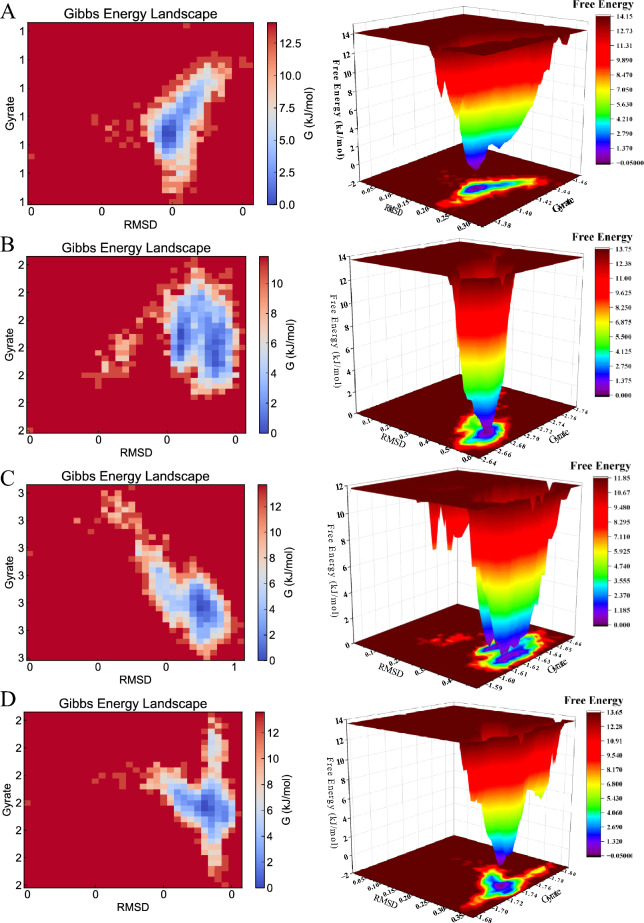
FEL of the four protein–ligand complexes based on RMSD and Rg. (**A)** AKT1–puerarin. (**B)** ALB–puerarin. (**C)** IL6–puerarin. (**D)** NFKB1–puerarin.

Based on RMSD and Rg data, Gibbs free energy landscape (FEL) plots were generated for all four complexes (Fig. 5A–D). In these maps, blue and purple regions represent low free-energy states associated with greater thermodynamic stability. Specifically, blue regions indicate the most stable conformations, whereas purple regions correspond to slightly less stable but energetically favorable states^36^.

The FEL plots of the AKT1–puerarin and ALB–puerarin complexes displayed concentrated low-energy basins, suggesting that puerarin binding imposes conformational constraints on the proteins and promotes stable equilibrium states. These results indicate that both complexes maintain structurally rigid and energetically favorable conformations during the simulations.

In contrast, the IL6–puerarin complex exhibited a broader and more dispersed energy distribution, forming a characteristic “wide valley” in the free energy landscape. This pattern indicates greater conformational heterogeneity and suggests dynamic transitions among multiple conformational states. Such flexibility may be attributed to the intrinsic structural plasticity of IL6, which contains flexible domains and participates in diverse ligand interactions. This conformational adaptability may be functionally relevant, given IL6’s role in immune regulation and signal transduction.

The NFKB1–puerarin complex showed an intermediate distribution pattern, with energy minima that were neither highly clustered nor widely dispersed, indicating moderate conformational flexibility. As a key transcription factor, NFKB1 typically undergoes structural rearrangements prior to DNA binding, and the observed FEL characteristics may reflect this functional adaptability. The free-energy distribution is therefore consistent with the known conformational dynamics of NFKB1 during transcriptional regulation.

### Binding free energy analysis

Lower binding free energy (ΔG_bind) values generally indicate stronger receptor–ligand affinity^37^. Using the MM-PBSA method, the calculated ΔG_bind values for the four protein–puerarin complexes are presented in Table 4 and Supplementary Figure S3A: AKT1–puerarin (−29.88 kcal/mol), ALB–puerarin (−27.28 kcal/mol), IL6–puerarin (−26.33 kcal/mol), and NFKB1–puerarin (−21.72 kcal/mol). Among these, the AKT1–puerarin complex exhibited the lowest binding free energy, indicating the most stable interaction. This result is consistent with the molecular docking and MD simulation findings.

**Table 4:** Energies between ligand and protein in complexes (kcal/mol).

Energies	Puerarin-AKT1	Puerarin-ALB	Puerarin-IL6	Puerarin-NFKB1
Van der Waals energy (VDWAALS)	−42.59	−39.93	−41.74	−30.49
Electrostatic energy (EEL)	0.00	−6.26	−7.85	−3.52
Polar solvation free energy (EPB)	12.88	21.98	26.12	14.69
Nonpolar solvation free energy (ENPOLAR)	−0.17	−3.07	−2.85	−2.40
Molecular mechanics term (GGAS)	−42.59	−46.19	−49.59	−34.01
Molecular solvation term (GSOLV)	12.72	18.91	23.26	12.29
Total binding free energy (TOTAL)	−29.88	−27.28	−26.33	−21.72

Energy decomposition at the residue level was further performed to identify amino acids contributing most significantly to ligand binding and complex stabilization. Key residues, including ILE-87, THR-356, ASN-63, LEU-165, PRO-221, and SER-223, showed major contributions to the binding free energy (Supplementary Figure S3B), highlighting their critical roles in the interaction networks between puerarin and AKT1, ALB, IL6, and NFKB1. Notably, these core residues may also be functionally associated with regulation of the TNF and IL-17 signaling pathways, suggesting their potential importance in puerarin-mediated signal transduction.

### Neuroprotective effect of puerarin against ferroptosis in HT22 cells injured by glutamate + erastin

**Fig. 6:**
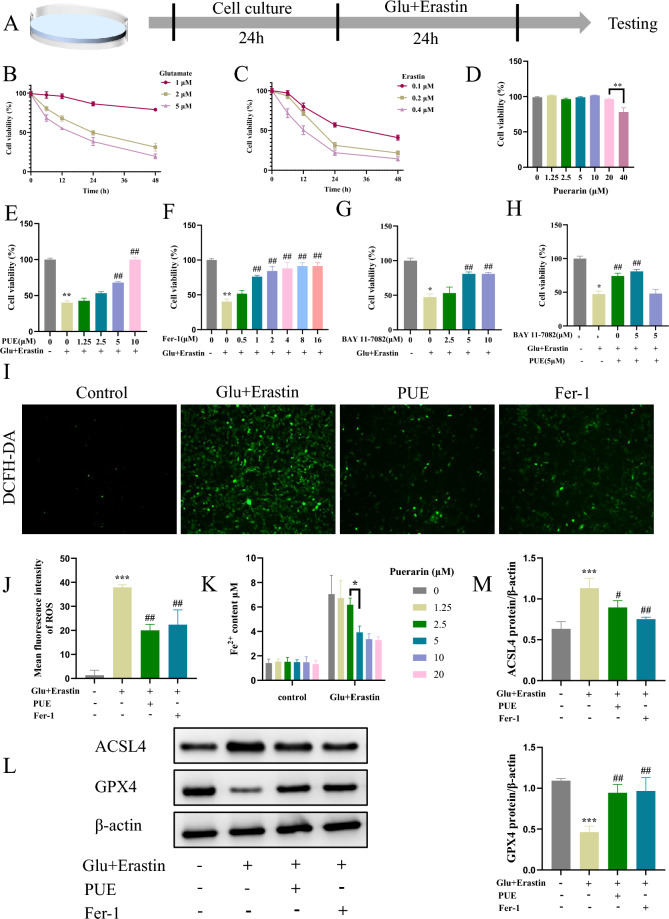
Puerarin’s inhibitory action on ferroptosis in HT22 cells induced by Glu + Erastin. (**A)** Schematic representation of the cellular experimental design. (**B-H)** Cell viability was assessed using the MTT assay to determine the optimal modeling conditions and intervention parameters. Specifically, panels show: optimization of glutamate and erastin exposure duration and concentration, determination of the optimal puerarin concentration, validation of the ferroptosis inhibitor Fer-1, and dose-dependent effects of the NF-κB inhibitor BAY 11-7082 (0, 2.5, 5, and 10 μM). In addition, the effects of BAY 11-7082 alone or in combination with puerarin on cell viability were evaluated. (**I)** Fluorescence images of ROS. (**J)** Graph of ROS content. (**K)** Intracellular Fe^2+^ content. (**L)** Western blot profile. Membranes were cut into sections prior to antibody incubation based on molecular weight; full-length blots are provided in Supplementary Raw Blots. (**M)** Expression levels of GPX4 and ACSL4 proteins. ^#^P<0.05, ^##^P<0.01 vs. control group; ^*^P<0.05, ^**^P<0.01 vs. Glu+Erastin group; n=3.

To investigate the neuroprotective mechanism of puerarin in TBI, an in vitro injury model was established by treating HT22 cells with glutamate and erastin (Fig. 6A). Four experimental groups were initially defined: control, injury (glutamate + erastin), puerarin intervention (10 μM), and ferroptosis inhibitor Fer-1 (8 μM). MTT assays (Fig. 6B–H) were performed to optimize modeling conditions and intervention parameters. Combined treatment with glutamate and erastin for 24 h reduced cell viability to approximately 50% of the control level, confirming successful model establishment. The optimal concentrations were determined to be 10 μM for puerarin and 8 μM for Fer-1. In addition, dose–response analysis of the NF-κB inhibitor BAY 11-7082 indicated that 5 μM effectively improved cell viability without inducing cytotoxicity, and was therefore selected for subsequent experiments.

To further explore the involvement of TNF signaling, HT22 cells were pretreated with BAY 11-7082 prior to ferroptosis induction. Notably, BAY 11-7082 significantly alleviated glutamate/erastin-induced cell injury, exhibiting a protective effect comparable to that of puerarin. Importantly, combined treatment with puerarin and BAY 11-7082 did not result in a further increase in cell viability, suggesting a lack of additive effect. These findings indicate that puerarin may exert its protective effects, at least in part, through modulation of the TNF signaling pathway.

ROS levels were assessed using the DCFH-DA fluorescent probe (Fig. 6I–J). The injury group exhibited markedly elevated ROS production, whereas both puerarin and Fer-1 significantly suppressed ROS accumulation. Consistently, intracellular Fe^2+^ levels were significantly increased following glutamate/erastin treatment and were effectively reduced by puerarin and Fer-1 (P < 0.05) (Fig. 6K), indicating inhibition of ferroptosis.

Western blot analysis (Fig. 6L–M) further demonstrated that puerarin markedly upregulated GPX4 expression while downregulating ACSL4 levels. These results suggest that puerarin alleviates ferroptosis by restoring redox homeostasis and modulating the ACSL4/GPX4 axis, potentially through regulation of TNF/NF-κB signaling.

### Puerarin improves glutamate/erastin-mediated morphological abnormalities of ferroptosis in HT22 cells

**Fig. 7:**
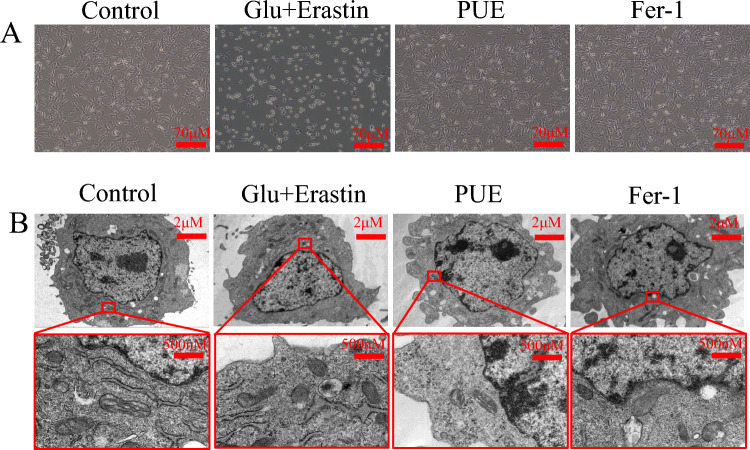
Puerarin alleviates morphological changes related to ferroptosis in HT22 cells. (**A)** Morphological characteristics of HT22 cells in different intervention groups visualized via a light microscope (scale bar = 70 μm). (**B)** Ultrastructural changes of mitochondria in cells of each group observed by transmission electron microscopy (scale bar = 2 μm and 500 nm).

Light microscopy observations showed that glutamate/erastin co-treatment significantly reduced synapse-like connections compared with the control group, with some cells exhibiting soma shrinkage and loss of neuron-like morphology. After treatment with 10 μM puerarin, intercellular connections became more compact, and the proportion of floating cells decreased (Fig. 7A).

Transmission electron microscopy further revealed that mitochondria in the model group displayed typical ferroptosis-related features, including reduced volume, decreased cristae, and increased membrane density. These ultrastructural abnormalities were markedly alleviated in the puerarin-treated group (Fig. 7B). These morphological findings confirm that puerarin effectively attenuates ferroptosis-associated cellular damage.

### Puerarin inhibits ferroptosis via the TNF signaling pathway

**Fig. 8:**
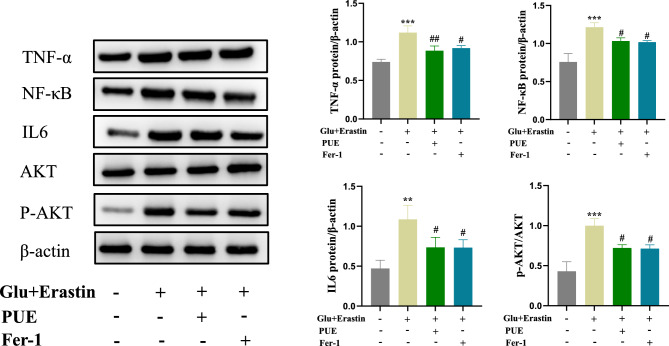
Regulatory effect of puerarin on the TNF signaling pathway in HT22 cells. (**A-B)** Expression quantities of TNF-α, NF-κB, IL-6, and p-AKT/AKT proteins in HT22 cells under different treatment conditions detected by Western blot. A 24-hour puerarin intervention group was set up, and β-actin was used as the internal reference protein for quantitative normalization (n=3). Membranes were cut into sections prior to antibody incubation based on molecular weight; full-length blots are provided in Supplementary Raw Blots. ^**^p<0.01 vs. control group; ^##^p<0.01, ^#^p<0.05 vs. glutamate + erastin group. Data are expressed as mean ± SEM.

To investigate whether the anti-ferroptotic effect of puerarin is associated with the TNF signaling pathway, protein expression levels were evaluated in the in vitro model. Western blot analysis showed that glutamate/erastin co-treatment significantly decreased the expression of TNF-α, NF-κB, IL-6, and p-AKT/AKT in HT22 cells (P < 0.01). Treatment with either puerarin or fer-1 markedly reversed these alterations (Fig. 8A–B). These results suggest that puerarin may inhibit ferroptosis by regulating key components of the TNF signaling pathway.

## Discussion

Ferroptosis is a distinct form of regulated cell death characterized by excessive phospholipid peroxidation, elevated ROS production, and mitochondrial dysfunction^38^. Neurons are particularly vulnerable to iron-dependent lipid peroxidation due to their membranes being enriched in polyunsaturated fatty acids (PUFAs) and cholesterol, rendering them highly susceptible to ferroptosis under conditions of oxidative stress^39^. Beyond the irreversible primary injury, TBI is typically accompanied by a cascade of secondary pathological events, including oxidative stress, inflammation, and blood–brain barrier disruption. These processes collectively disturb iron homeostasis, leading to intracellular iron accumulation, enhanced ROS generation, and subsequent activation of ferroptosis. This, in turn, exacerbates neuronal dysfunction through disruption of mitochondrial membrane potential and dysregulation of lipid metabolism^40^^,^^41^. Although ferroptosis has emerged as a critical contributor to TBI pathophysiology, its precise molecular mechanisms remain incompletely defined, and effective therapeutic strategies targeting this process are still lacking.

According to the Shennong Bencao Jing, Pueraria is traditionally described as having effects such as “quenching thirst and reducing internal heat.” Puerarin, its principal bioactive constituent, has been reported to attenuate ferroptosis by reducing intracellular iron accumulation, suppressing lipid peroxidation, and ameliorating mitochondrial dysfunction^42^^,^^43^. However, the molecular basis by which puerarin regulates ferroptosis in the context of TBI has yet to be fully elucidated. Therefore, investigating the anti-inflammatory and neuroprotective effects of puerarin through modulation of ferroptosis may provide both mechanistic insights and a theoretical foundation for the development of novel therapeutic strategies for TBI.

It should be noted that this study is based on a single TBI transcriptomic dataset (GSE58483), which introduces certain limitations. However, GSE58483 remains one of the few available datasets containing TBI-related transcriptomic data suitable for this type of analysis. Moreover, the integration of network pharmacology, molecular docking, and in vitro experimental validation enhances the reliability of the identified core targets. Network pharmacology analysis was conducted to identify shared core targets among puerarin, ferroptosis, and TBI. Five key targets—AKT1, ALB, IL6, NFKB1, and TNF—were identified. These targets exhibit distinct roles in ferroptosis regulation: IL6, NFKB1, and TNF function as drivers of ferroptosis, whereas AKT1 appears to exert an inhibitory effect. ALB, an antioxidant protein, participates in the indirect regulation of iron homeostasis. Specifically, AKT1 inhibits ferroptosis by stabilizing GPX4 expression and reducing ROS production^44^. IL6 promotes MARCH3 expression in hepatocytes, thereby accelerating GPX4 ubiquitination and degradation, which contributes to ferroptosis and lipid accumulation^45^. Activation of NF-κB upregulates ferroptosis-related genes, including NOX4 and ACSL4, thereby increasing ferroptosis sensitivity^46^. In addition, the TNF-α/TNFR1 signaling axis facilitates ferroptosis through ROS accumulation, whereas ferroptosis inhibitors can reduce excessive ROS production in TNF-α-treated cells and simultaneously promote osteogenesis and angiogenesis^47^. ALB mitigates iron overload and decreases free iron levels through its dual functions of metal chelation and antioxidant activity^48^. Collectively, these functional distinctions suggest that puerarin may modulate ferroptosis through the coordinated regulation of multiple targets.

KEGG enrichment analysis further indicated that the principal pathways through which puerarin ameliorates TBI via ferroptosis regulation include the lipid and atherosclerosis pathway, the TNF signaling pathway, and the IL-17 signaling pathway. Among these, lipid metabolism disorders are closely associated with neuroinflammation, apoptosis, and blood–brain barrier disruption following TBI. Aberrant activation of atherosclerosis-related pathways in brain tissue may contribute to vascular endothelial injury and dysfunction of the neurovascular unit^49^. Puerarin is therefore likely to preserve cerebral microvascular integrity by modulating lipid metabolism and suppressing inflammatory plaque formation, thereby facilitating a microenvironment conducive to neural repair^50^. The TNF signaling pathway is markedly activated during the acute phase of TBI and represents a central mediator of neuronal apoptosis and inflammatory cascades^51^. TNF-α can further activate the NF-κB and MAPK pathways, promoting the expression of pro-inflammatory cytokines such as IL-1β and IL-6 and exacerbating neuronal damage^52^. It is thus plausible that puerarin attenuates neuroinflammation and secondary injury by suppressing TNF pathway activity and reducing pro-inflammatory mediator levels. In parallel, abnormal activation of the IL-17 signaling pathway after TBI suggests that puerarin may exert therapeutic effects through coordinated modulation of multiple inflammatory pathways^53^.

Molecular docking analysis demonstrated that puerarin exhibits favorable binding affinities toward AKT1, ALB, IL6, and NFKB1. Integrating these findings with prior evidence, the neuroprotective mechanisms of puerarin can be summarized as follows: it suppresses neuroinflammation by modulating AKT1 signaling in microglia, inhibiting NF-κB activation and NLRP3 inflammasome activity, and reducing IL-6-mediated inflammatory responses^54^^,^^55^; it alleviates neuronal apoptosis via activation of the PI3K/AKT pathway, thereby improving neurological outcomes after TBI^56^; additionally, it exerts anti-inflammatory, antioxidant, and anti-apoptotic effects, partly associated with ALB-related regulatory functions^57^^,^^58^. Molecular dynamics simulations further confirmed that the puerarin–target complexes exhibit stable binding conformations, supporting the durability and specificity of these interactions.

To further validate the role of puerarin in modulating TBI-associated ferroptosis, in vitro experiments were performed. Although the glutamate/erastin-induced HT22 cell model does not fully recapitulate the complexity of in vivo TBI, it provides a well-established platform for investigating ferroptosis-related neuronal injury. In this study, HT22 cells were co-treated with glutamate and erastin to induce ferroptosis^59^, while Fer-1 was used as a positive control. MTT assay results showed that puerarin significantly improved cell viability following 24 h of glutamate/erastin exposure. Furthermore, intracellular ROS and Fe^2+^ levels were markedly elevated in the injury model, whereas puerarin treatment effectively reduced their accumulation, comparable to the effects of Fer-1, indicating a potential anti-ferroptotic role. Western blot analysis further demonstrated that puerarin upregulated GPX4 expression while downregulating ACSL4 levels. As a key inhibitor of lipid peroxidation, GPX4 suppresses ferroptosis, whereas ACSL4 enhances ferroptosis sensitivity by promoting polyunsaturated fatty acid metabolism^60^. These findings suggest that puerarin restores iron homeostasis and inhibits lipid peroxidation through modulation of the ACSL4/GPX4 axis, thereby attenuating ferroptosis progression.

To investigate the involvement of TNF signaling, pharmacological inhibition using the NF-κB inhibitor BAY 11-7082 was performed. BAY 11-7082 significantly alleviated glutamate/erastin-induced cell injury, showing a protective effect comparable to that of puerarin. Notably, combined treatment with puerarin and BAY 11-7082 did not produce a further enhancement of cell viability, suggesting a lack of additive effect. This observation indicates that puerarin and NF-κB inhibition may act, at least in part, through a shared signaling pathway. These findings provide experimental support for the involvement of the TNF/NF-κB axis in the regulation of ferroptosis by puerarin.

Morphological observations provided additional support. Under light microscopy, puerarin ameliorated pathological features such as synaptic loss and cellular shrinkage. Transmission electron microscopy further revealed that puerarin reversed ferroptosis-associated ultrastructural alterations, including mitochondrial shrinkage and reduced cristae density^61^. These results corroborate the anti-ferroptotic effects of puerarin at the structural level. Consistent with previous studies, TBI induces iron overload and lipid metabolic disturbances in brain tissue^62^. Disrupted iron homeostasis increases intracellular Fe^2+^, which promotes ROS generation via the Fenton reaction and accelerates lipid peroxidation^63^. Moreover, dysregulated expression of ferroptosis-related genes, including GPX4 and ACSL4, has been reported in TBI^64^, consistent with the findings of the present study.

**Fig. 9:**
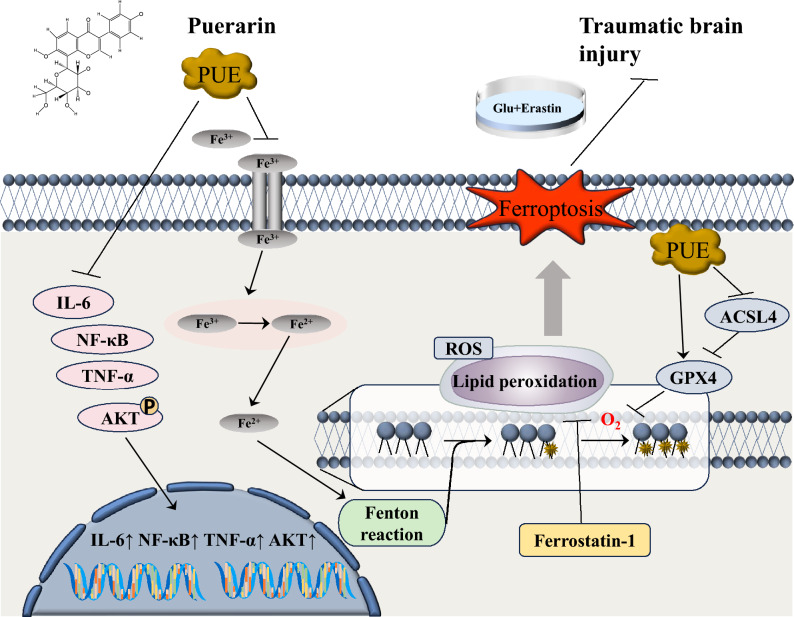
Puerarin inhibits ferroptosis through multiple mechanisms. In the glutamate/erastin-induced HT22 cell model, puerarin exerts neuroprotective effects through multiple mechanisms. Puerarin upregulates GPX4 expression and downregulates ACSL4, thereby reducing lipid peroxidation. In addition, puerarin decreases intracellular ROS and Fe^2+^ accumulation, contributing to the suppression of ferroptosis. Pharmacological inhibition experiments further suggest that puerarin may modulate ferroptosis, at least in part, through the TNF signaling pathway. Through coordinated regulation of oxidative stress, lipid metabolism, and inflammatory signaling, puerarin ultimately alleviates ferroptotic neuronal injury.

Although not exclusively ferroptosis-specific, the identified GO terms, KEGG pathways, and core targets (TNF, IL6, AKT1, NFKB1, HIF1A, PPARG) are closely associated with ferroptosis regulation, suggesting that puerarin may modulate ferroptosis through an upstream multi-target network. Notably, the anti-ferroptotic effects of puerarin appear to be closely associated with TNF signaling. Western blot results showed that glutamate/erastin treatment significantly altered the expression of TNF-α, NF-κB, IL-6, and p-AKT/AKT, whereas puerarin and Fer-1 partially reversed these changes. Combined with the results of pathway inhibition experiments, these findings suggest that puerarin may coordinately regulate ferroptosis-related processes by modulating key components of the TNF signaling pathway, thereby exerting neuroprotective effects. The proposed mechanism is illustrated in Fig. 9.

This study has several limitations. First, network pharmacology and molecular docking analyses depend on existing databases and literature, which may introduce bias. Second, experimental validation was limited to in vitro models, and although pharmacological inhibition experiments provided supportive evidence, more direct approaches (such as genetic manipulation or in vivo validation) are required to confirm the causal role of TNF signaling. Future studies should incorporate multi-omics datasets, animal models, and pathway-specific interventions to further elucidate the underlying mechanisms of puerarin in TBI.

## Conclusion

This study integrates network pharmacology, molecular docking, molecular dynamics simulations, and experimental validation to systematically investigate the therapeutic potential of puerarin in TBI. The findings demonstrate that puerarin exerts neuroprotective effects by inhibiting ferroptosis and regulating oxidative stress and iron metabolism. Mechanistically, puerarin appears to modulate key signaling pathways, particularly the TNF/NF-κB axis, and coordinately regulate core targets such as AKT1, IL6, and NFKB1. These results provide experimental evidence supporting the involvement of TNF-related signaling in the anti-ferroptotic effects of puerarin and offer a theoretical basis for the development of novel therapeutic strategies for TBI.

## Supplementary Information

Supplementary Information 1.

Supplementary Information 2.

Supplementary Information 3.

Supplementary Information 4.

Supplementary Information 5.

Supplementary Information 6.

Supplementary Information 7.

## Data Availability

The data are publicly available and can be found within the article.
