# Supplementary material for: Network pharmacology analysis and cellular experiment validation of puerarin in attenuating TBI through suppression of ferroptosis
Source: Sci Rep. 2026 May 25;16:23810. doi: 10.1038/s41598-026-53728-5 (PMC13434821; doi:10.1038/s41598-026-53728-5)
Supplement: Supplementary file 1 [file 41598_2026_53728_MOESM1_ESM.pdf]

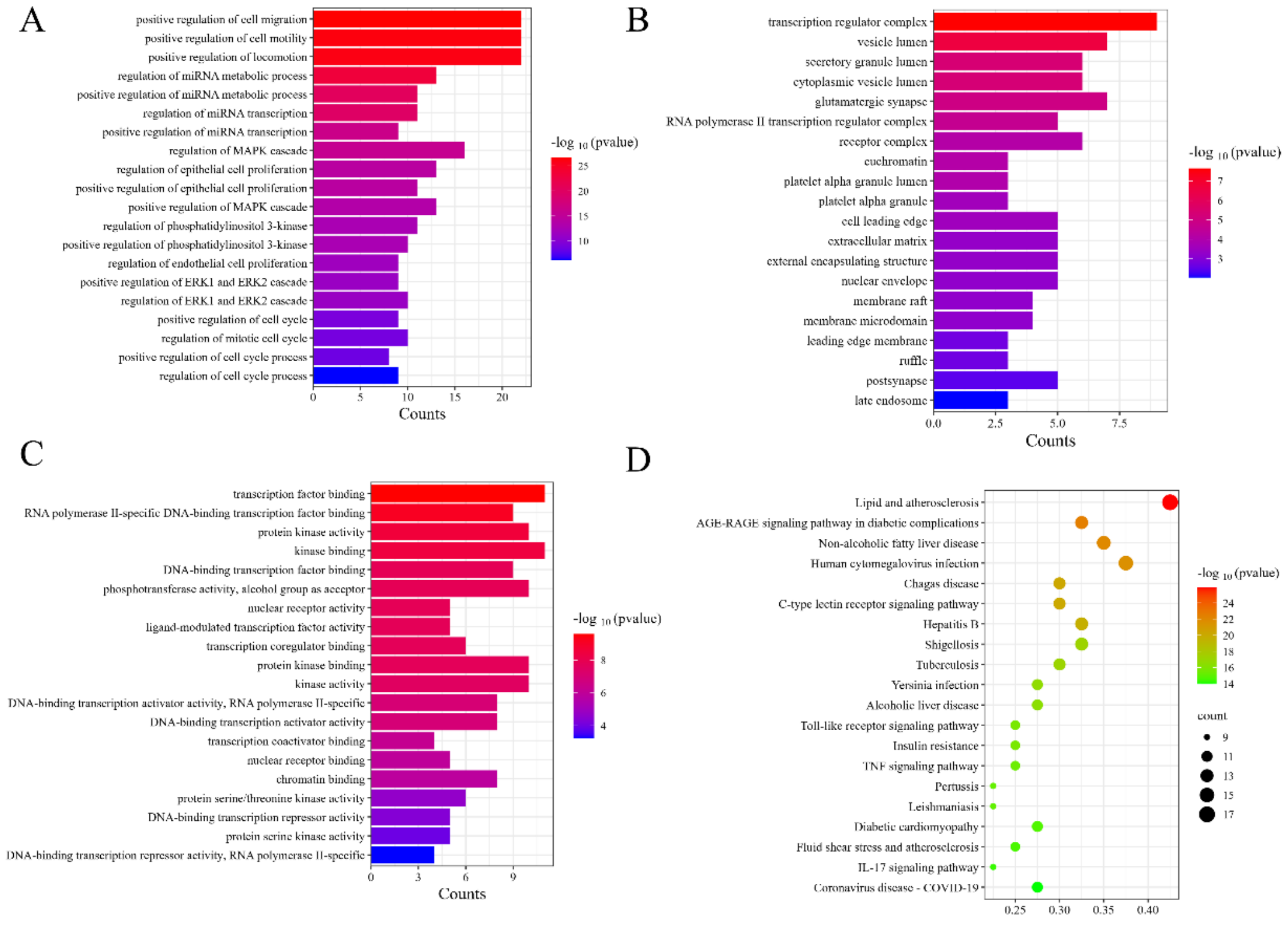

Supplementary Figure S1. GO and KEGG analysis of ferroptosis-related overlapping targets. **A** Top 20 terms in the BP. **B** The top 20 terms within the CC. **C** The top 20 terms within the MF. **D** KEGG pathway enrichment analysis bubble plot.
