# Supplementary material for: Network pharmacology analysis and cellular experiment validation of puerarin in attenuating TBI through suppression of ferroptosis
Source: Sci Rep. 2026 May 25;16:23810. doi: 10.1038/s41598-026-53728-5 (PMC13434821; doi:10.1038/s41598-026-53728-5)
Supplement: Supplementary file 2 [file 41598_2026_53728_MOESM2_ESM.pdf]

A

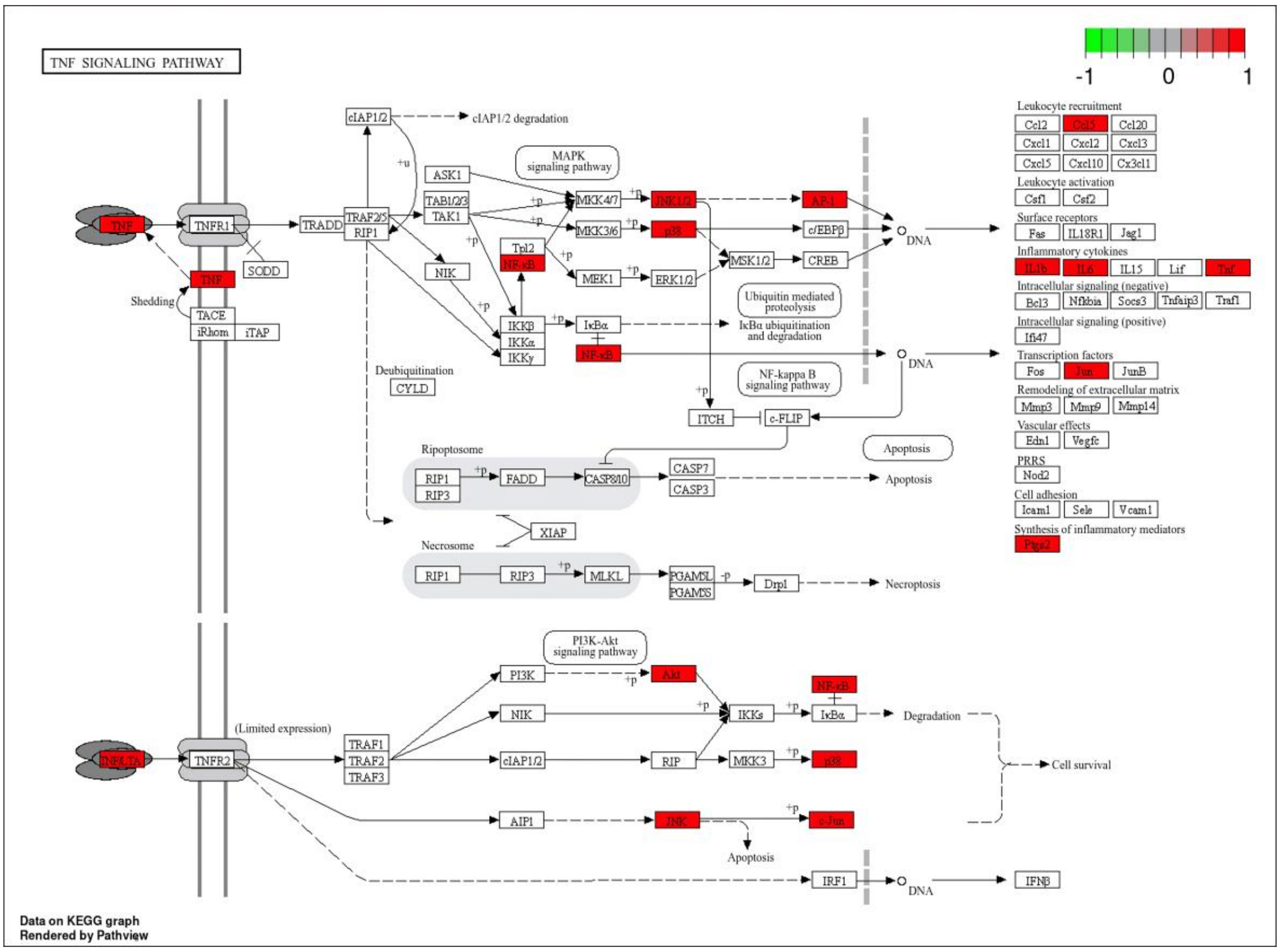

B

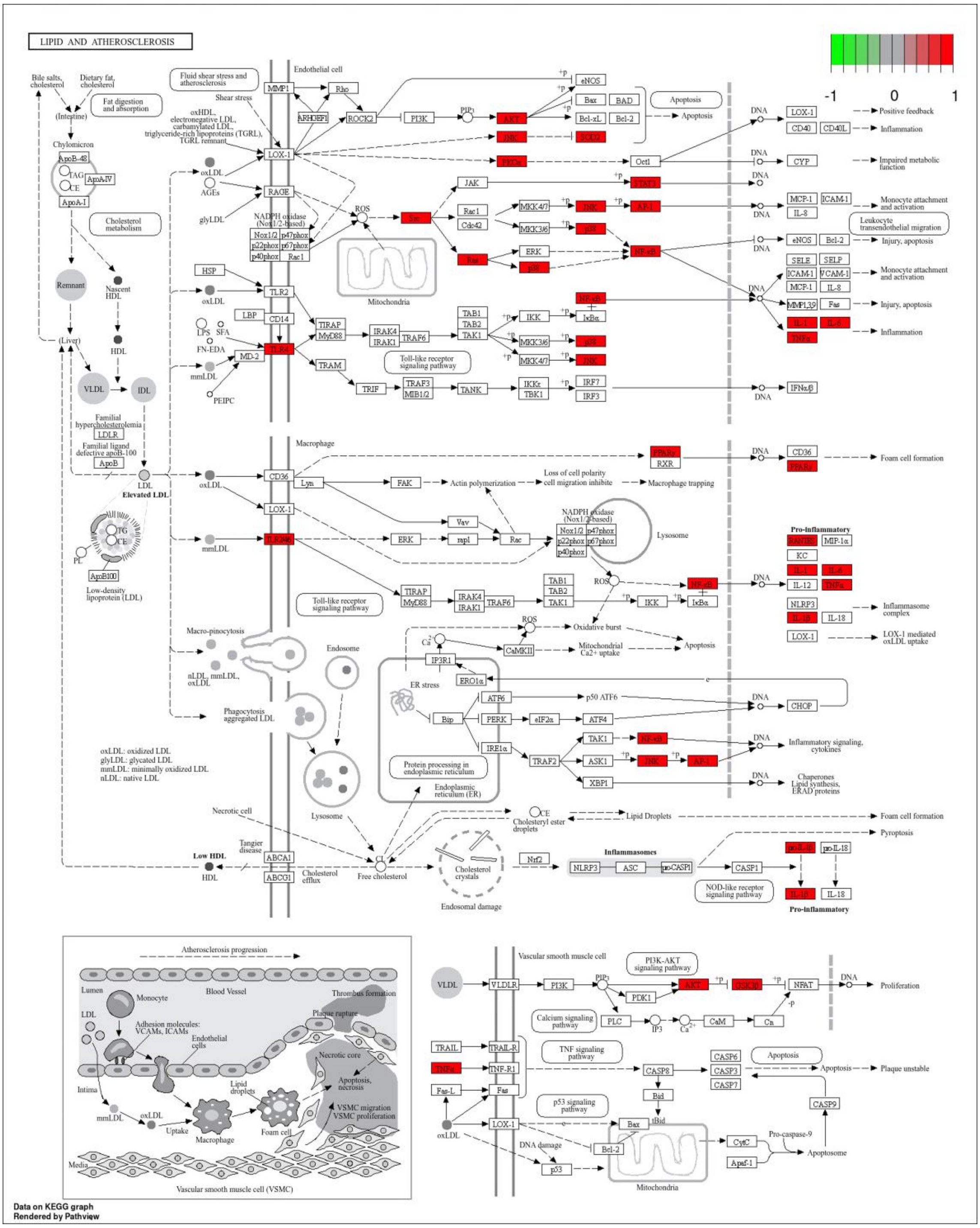

Supplementary Figure S2. A Lipid and atherosclerosis pathway. **B** TNF signaling pathway. Red-labeled nodes represent potential targets of puerarin. Pathway map obtained from KEGG (Kyoto Encyclopedia of Genes and Genomes, https://www.kegg.jp/). © Kanehisa Laboratories.
