# Supplementary material for: Network pharmacology analysis and cellular experiment validation of puerarin in attenuating TBI through suppression of ferroptosis
Source: Sci Rep. 2026 May 25;16:23810. doi: 10.1038/s41598-026-53728-5 (PMC13434821; doi:10.1038/s41598-026-53728-5)
Supplement: Supplementary file 3 [file 41598_2026_53728_MOESM3_ESM.pdf]

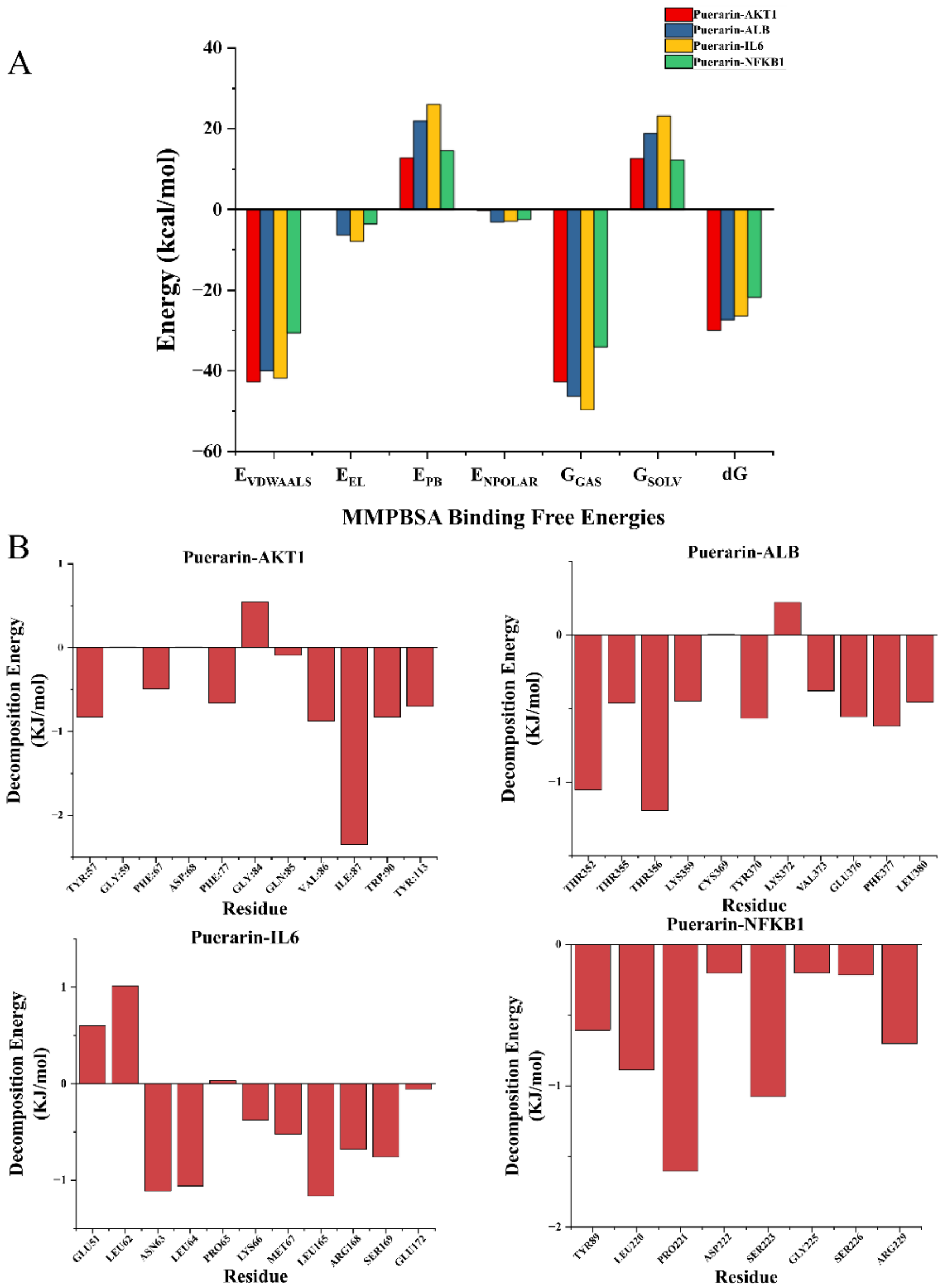


**Supplementary Figure S3. A** The binding free energy calculated from molecular dynamics simulations. **B** The contribution of each residue to the energy was evaluated using per-residue decomposition analysis.
