# Supplementary material for: Network pharmacology analysis and cellular experiment validation of puerarin in attenuating TBI through suppression of ferroptosis
Source: Sci Rep. 2026 May 25;16:23810. doi: 10.1038/s41598-026-53728-5 (PMC13434821; doi:10.1038/s41598-026-53728-5)
Supplement: Supplementary file 4 [file 41598_2026_53728_MOESM4_ESM.pdf]

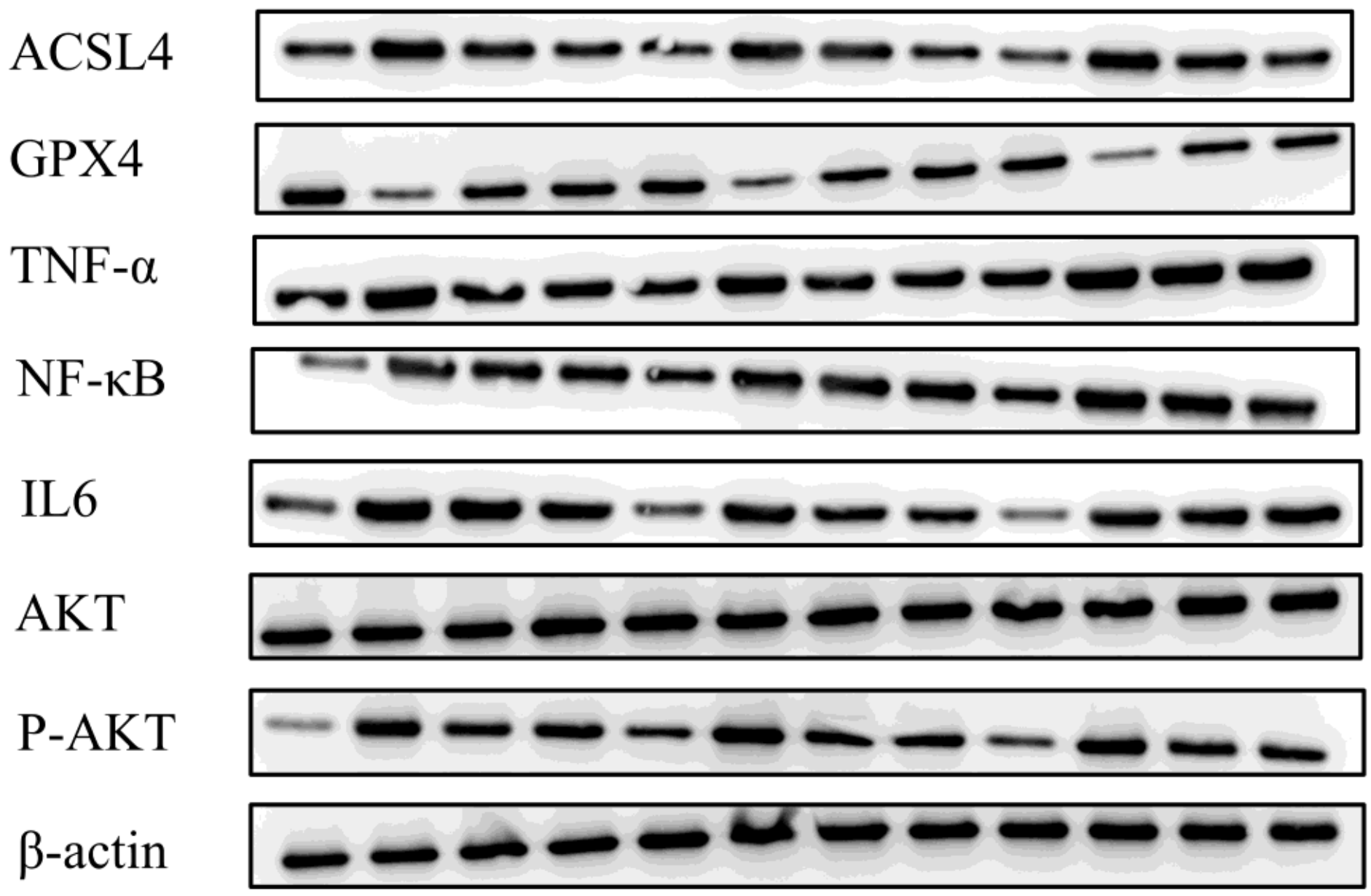

Supplementary Figure S4. WB Experimental Raw Data
