# Supplementary material for: Network pharmacology analysis and cellular experiment validation of puerarin in attenuating TBI through suppression of ferroptosis
Source: Sci Rep. 2026 May 25;16:23810. doi: 10.1038/s41598-026-53728-5 (PMC13434821; doi:10.1038/s41598-026-53728-5)
Supplement: Supplementary file 5 [file 41598_2026_53728_MOESM6_ESM.pdf]

Supplementary Table S1 Topological parameters of the hub genes identified in the PPI network.

Name	BetweennessCentrality	ClosenessCentrality	Degree
ALB	0.031154805636375568	0.9512195121951219	37
TNF	0.027055647184990397	0.9285714285714286	36
AKT1	0.028485110658988278	0.9285714285714286	36
IL6	0.021421904709142653	0.9069767441860465	35
PPARG	0.023367370774041912	0.9069767441860465	35
TGFB1	0.028802044995143983	0.9069767441860465	35
HIF1A	0.016944357215400826	0.8863636363636364	34
JUN	0.012554010719791172	0.8666666666666667	33
PTGS2	0.012970122573149988	0.8666666666666667	33
NFKB1	0.01334849663573458	0.8666666666666667	33
