# Supplementary material for: Network pharmacology analysis and cellular experiment validation of puerarin in attenuating TBI through suppression of ferroptosis
Source: Sci Rep. 2026 May 25;16:23810. doi: 10.1038/s41598-026-53728-5 (PMC13434821; doi:10.1038/s41598-026-53728-5)

Supplementary_Raw_Blots

Due to differences in target molecular weights, membranes were cut into appropriate sections prior to antibody incubation. Therefore, full-length blots are not available. All provided images represent the original, unprocessed membrane sections used for detection without selective cropping.

Fig6_WB_raw_ACSL4

80 kDa ACSL4

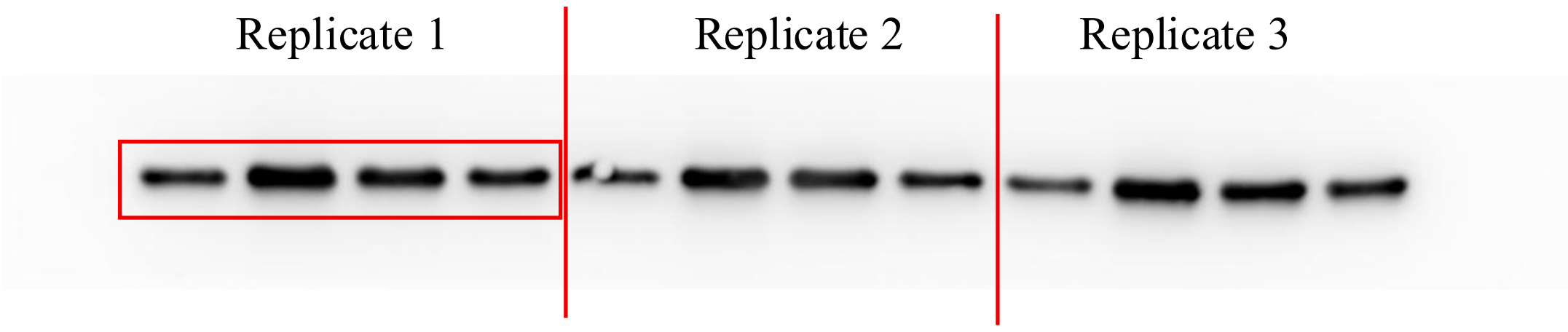

Fig6_WB_raw_GPX4

20 kDa GPX4

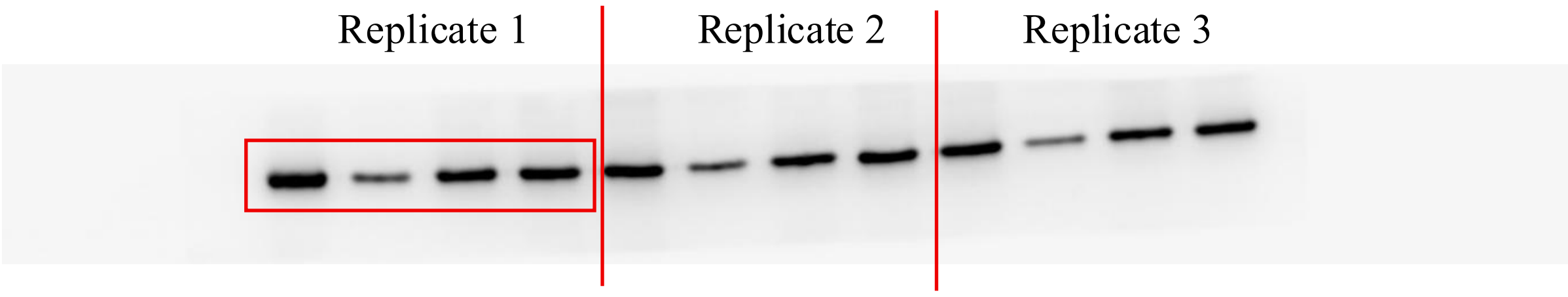

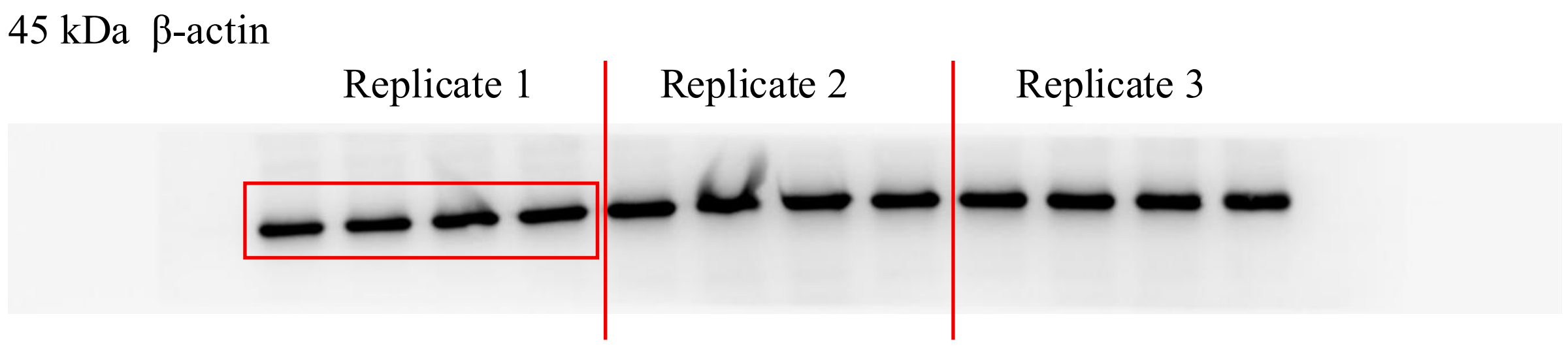
45 kDa β-actin
Replicate 1
Replicate 2
Replicate 3

Fig8_WB_raw_TNF-α

25 kDa TNF-α

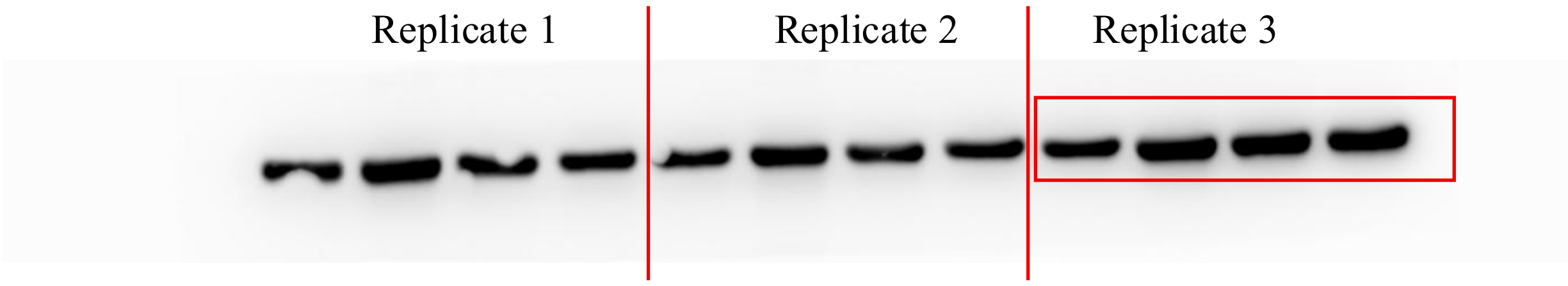

65 kDa NF-κB

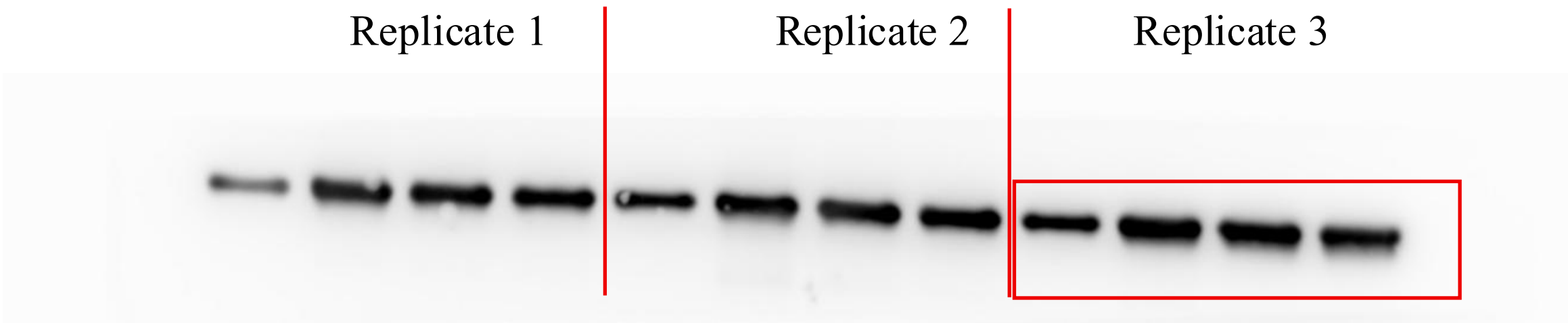

Fig8_WB_raw_IL6

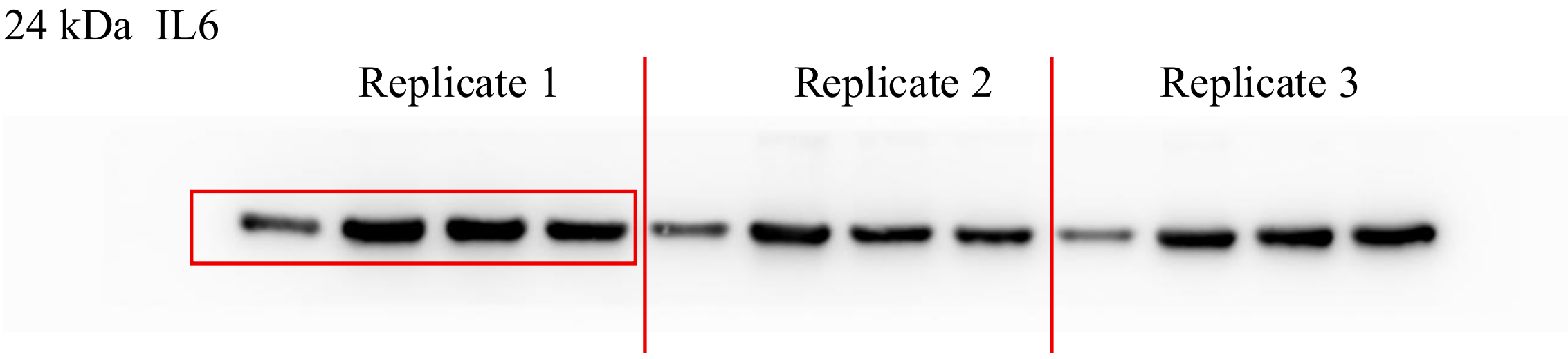

60 kDa AKT

Replicate 1 Replicate 2 Replicate 3

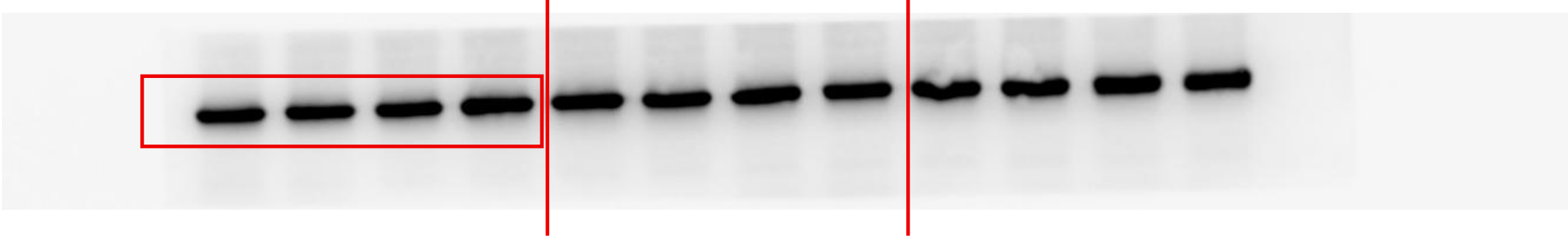

Fig8_WB_raw_ P-AKT

60 kDa P-AKT

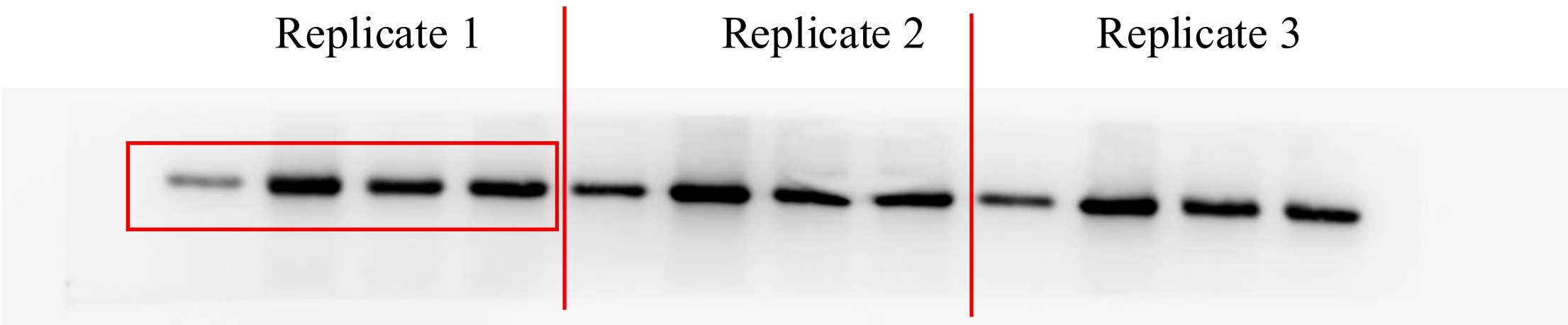

Fig8_WB_raw_β-actin

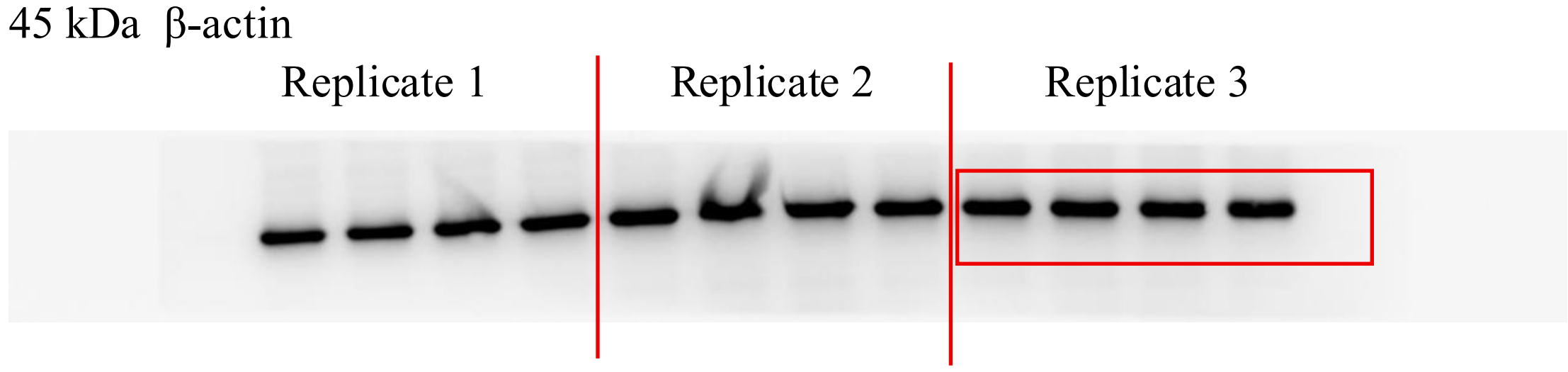

Supplement: Supplementary file 6 [file 41598_2026_53728_MOESM7_ESM.pdf]
